# A Technology-Based Pregnancy Health and Wellness Intervention (Two Happy Hearts): Case Study

**DOI:** 10.2196/30991

**Published:** 2021-11-17

**Authors:** Tamara Jimah, Holly Borg, Priscilla Kehoe, Pamela Pimentel, Arlene Turner, Sina Labbaf, Milad Asgari Mehrabadi, Amir M. Rahmani, Nikil Dutt, Yuqing Guo

**Affiliations:** 1 Sue & Bill Gross School of Nursing University of California, Irvine Irvine, CA United States; 2 First 5 Orange County Children & Families Commission Santa Ana, CA United States; 3 Department of Computer Science University of California, Irvine Irvine, CA United States; 4 Department of Electrical Engineering and Computer Science University of California, Irvine Irvine, CA United States; 5 Institute for Future Health University of California, Irvine Irvine, CA United States

**Keywords:** ecological momentary assessment, heart rate, mHealth, physical activity, pregnancy, sleep, wearable electronic device

## Abstract

**Background:**

The physical and emotional well-being of women is critical for healthy pregnancy and birth outcomes. The Two Happy Hearts intervention is a personalized mind-body program coached by community health workers that includes monitoring and reflecting on personal health, as well as practicing stress management strategies such as mindful breathing and movement.

**Objective:**

The aims of this study are to (1) test the daily use of a wearable device to objectively measure physical and emotional well-being along with subjective assessments during pregnancy, and (2) explore the user’s engagement with the Two Happy Hearts intervention prototype, as well as understand their experiences with various intervention components.

**Methods:**

A case study with a mixed design was used. We recruited a 29-year-old woman at 33 weeks of gestation with a singleton pregnancy. She had no medical complications or physical restrictions, and she was enrolled in the Medi-Cal public health insurance plan. The participant engaged in the Two Happy Hearts intervention prototype from her third trimester until delivery. The *Oura* smart ring was used to continuously monitor objective physical and emotional states, such as resting heart rate, resting heart rate variability, sleep, and physical activity. In addition, the participant self-reported her physical and emotional health using the Two Happy Hearts mobile app–based 24-hour recall surveys (sleep quality and level of physical activity) and ecological momentary assessment (positive and negative emotions), as well as the Perceived Stress Scale, Center for Epidemiologic Studies Depression Scale, and State-Trait Anxiety Inventory. Engagement with the Two Happy Hearts intervention was recorded via both the smart ring and phone app, and user experiences were collected via Research Electronic Data Capture satisfaction surveys. Objective data from the *Oura* ring and subjective data on physical and emotional health were described. Regression plots and Pearson correlations between the objective and subjective data were presented, and content analysis was performed for the qualitative data.

**Results:**

Decreased resting heart rate was significantly correlated with increased heart rate variability (*r=*–0.92, *P*<.001). We found significant associations between self-reported responses and *Oura* ring measures: (1) positive emotions and heart rate variability (*r=0*.54, *P*<.001), (2) sleep quality and sleep score (*r*=0.52, *P<*.001), and (3) physical activity and step count (*r*=0.77, *P*<.001). In addition, deep sleep appeared to increase as light and rapid eye movement sleep decreased. The psychological measures of stress, depression, and anxiety appeared to decrease from baseline to post intervention. Furthermore, the participant had a high completion rate of the components of the Two Happy Hearts intervention prototype and shared several positive experiences, such as an increased self-efficacy and a normal delivery.

**Conclusions:**

The Two Happy Hearts intervention prototype shows promise for potential use by underserved pregnant women.

## Introduction

The pregnancy period is characterized by physiological and psychological changes, including those affecting cardiovascular response, hormonal balance, and sleep quality [1,2]. For instance, marked cardiovascular and autonomic nervous system adaptations result in various changes, such as increased heart rate [3]. Moreover, various studies have reported a decline in sleep quality, including shorter sleep duration and increased awakenings, during pregnancy as compared to the prepregnancy period, particularly among women in the third trimester [4-6]. In addition, physical activity levels are reduced during pregnancy, and these changes are more pronounced in the third trimester [7]. All these physiological changes together may make pregnancy a stressful period, and consequently impact the emotional well-being of pregnant women. Approximately 20% of pregnant women in the United States experience prenatal distress (ie, stress, depression, or anxiety) [8], with as high as 35% experiencing elevated distress during the COVID-19 pandemic [9]. It is important to recognize that women of low socioeconomic status are at a high risk of prenatal stress [10]. Hence, effective strategies are required to promote physical and emotional well-being, healthy pregnancies, and improved birth outcomes in these vulnerable populations.

Growing research has shown that physical activity and/or mindful breathing promote positive maternal health [11-14]. However, there are mixed results concerning regular physical activity and its benefits for improving sleep and emotional well-being. A meta-analysis reported that regular exercise during pregnancy reduced the odds of self-reported prenatal depression by 67% (but not anxiety levels), with a greater reduction observed among women receiving supervision during exercise [11]. Moreover, an integrated mind-body approach such as yoga has demonstrated a decrease in perceived stress, depression, and anxiety [15]. An objective indicator of emotional state as measured by heart rate variability (HRV) is the result of cardiovascular and neurological automatic regulation [16]. Previous meta-analyses provide inconsistent evidence for the physiological impact of physical activity on HRV and sleep among pregnant women [17]. Sleep comprises an important predictor of physical and mental health [18]. Recent studies show that regular physical activity improves self-reported sleep during pregnancy, but with varying effect sizes [17,19]. Although evidence suggests a decrease in perceived prenatal distress after completion of mindful breathing practices [12,13,20], little is known about the impact of mindful breathing on sleep during pregnancy.

Existing literature suggests that mind-body interventions play an essential role in health promotion through enhanced adaptive responses during pregnancy [15]. Furthermore, coaching and social support may enhance the impact of this integrated approach to achieve emotional well-being. In line with these findings, we created the Two Happy Hearts (THH) intervention based on the self-management theory that emphasizes an individual’s active engagement in self-monitoring and reflection and, subsequently, taking steps to develop healthy behaviors [21,22]. The THH intervention is a personalized mind-body program coached by community health workers (CHWs); it includes monitoring and reflecting on personal health, as well as practicing stress management strategies, such as mindful breathing and movement. CHWs play a critical role in promoting a healthy lifestyle for pregnant women by providing compassionate listening, connecting women to community resources, and setting personalized goals and action plans tailored to their capacity and need, while supporting them to overcome potential barriers [23]. By building self-efficacy through developing and sustaining healthy habits, THH empowers women to gain self-management skills to proactively cope with stress, anxiety, and depression during pregnancy.

Self-management can be enhanced with the adoption of wearable internet of things (WIoT) technology [24]. Wearable sensors have been used to objectively assess maternal health and well-being during pregnancy given their validity and ubiquitous health monitoring [25-27]. In our study, we used the *Oura* ring, a smart ring validated to detect sleep characteristics and quality [28]. Previous research has confirmed the validity of the ring in measuring resting heart rate and HRV [29], as well as a reliable method of quantifying the level of physical activity via step count [30]. We used the *Oura* ring to collect objective data to complement self-reported responses to help pregnant women monitor their physical and emotional well-being, and to track the completion rate of the THH mindful breathing and movement components. The overarching research question was: What is the participant’s usage and her perspectives regarding the THH intervention prototype? The specific aims of the study were to (1) test the daily use of a wearable device to objectively measure physical and emotional well-being along with subjective assessments during pregnancy, and (2) explore the user’s engagement with the THH intervention prototype, as well as understand their experiences with the prototype components.

## Methods

### Study Design

This case study design involved a single participant receiving the THH intervention prototype. Both quantitative and qualitative data were collected using a wearable device and by administering closed- and open-ended survey questionnaires.

### THH Intervention Prototype

We developed the THH intervention prototype according to the American College of Obstetricians and Gynecologists (ACOG) exercise guidelines and updated evidence on mindful breathing [12,31,32]. Mindful breathing involved paced deep breathing (ie, 3 minutes per practice, twice a day). Mindful movement included brisk walking (ie, 30 minutes on most days), and strength movement (ie, squat, plie squat, wall push-up, one-arm row, bicep curl, overhead press, and low row) with 12 to 15 repetitions per movement (3 times per week). Walking and strength movement started and ended with 2 minutes of paced deep breathing. All study visits were conducted virtually by a trained CHW. The CHW assessed the participant’s physical and mental health history, helped set personalized goals, created an action plan suitable for the participant’s health condition, fitness level and schedule, as well as provided continuous support via text messages and/or brief phone conversations.

**Figure 1:**
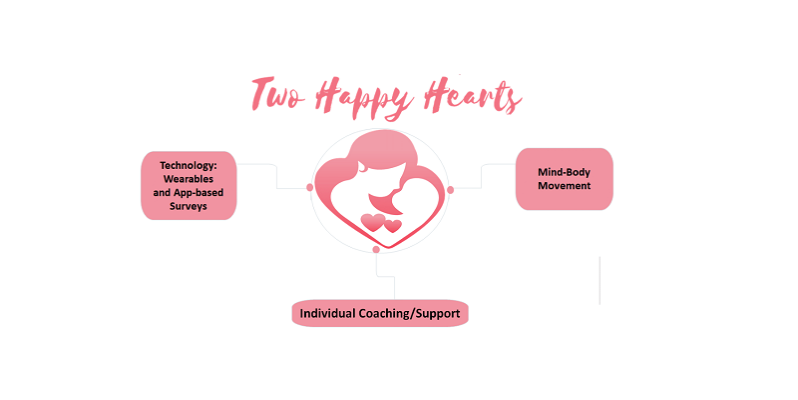
Components of the Two Happy Hearts intervention prototype.

A unique feature of the THH intervention prototype was the leveraging of technology to strengthen emotional and social support provided to the participant. The *Oura* ring was used to enhance the participant’s awareness of self-monitoring of vital health indicators, such as resting heart rate, resting HRV, sleep, and physical activity. Moreover, our research team designed the THH mobile phone app to enhance the participant’s experience with the intervention. The THH app was developed using ZotCare, a health cybernetic service platform that makes it possible to build a cybernetic system for data collection, labeling, and intervention. Specifically, the app-based surveys helped with self-reflection on physical and emotional well-being. In addition, video demonstrations and guidelines were incorporated into the app to increase the user’s access to health-related education material on mindful breathing and movement. Figure 1 shows the different components of the THH intervention prototype.

### Study Setting and Participant Recruitment

We recruited 1 low-income pregnant woman from MOMS Orange County (MOMS OC), a community-based nonprofit organization that supports underserved perinatal women in Orange County, California. The participant received the study flyer from MOMS OC and contacted the THH research team. She was subsequently screened by the study coordinator for the following eligibility criteria: adult aged 18-40 years, having a singleton pregnancy without medical complications restricting physical activity, not engaged in a coached physical activity and/or mindfulness program, and having a smartphone. The participant consented to engage in the THH intervention prototype facilitated by a CHW virtually, as required per COVID-19 pandemic regulations.

### Data Collection

#### Quantitative Data

Objective data were obtained from the *Oura* ring. Subjective data (ie, responses to closed-ended questions) were collected via the app-based surveys and the Research Electronic Data Capture (REDCap), a secure web-based data collection survey tool [33]. The app-based surveys included sleep recall, physical activity recall, and ecological momentary assessment (EMA). The REDCap collected pre-post intervention surveys at 33 weeks of gestation, before the start of the intervention, and at 1 week post partum.

#### Qualitative Data

Qualitative data were obtained from open-ended questions about the participant’s user experience with the THH intervention. The CHW recorded the participant’s responses on REDCap during the interview. Additional feedback was received from the participant via email.

### Measures

#### Objective Measures of Physical and Emotional Well-being

For the objective data, we used within-subject repeated measures to monitor physical and emotional well-being. Cardiovascular response (ie, average resting heart rate and HRV), sleep, and physical activity data were collected daily using the *Oura* ring [34]. Resting HRV was calculated in terms of the root mean square of successive difference (RMSSD) of interbeat intervals. Two attributes of sleep were selected for our analysis: sleep stages and sleep score. Sleep stages include deep, rapid eye movement (REM), and light sleep. Sleep score ranges from 0 to 100 and denotes the quality of sleep [34]. The following are suggested sleep score ranges to determine sleep quality: <70 (pay attention to sleep); ≥70 to 84 (good); and ≥85 (optimal) [34]. Step-count data from the ring was used as an indicator of physical activity level. The frequency and duration of the THH mindful breathing and strength movement practice were recorded with the THH mobile app.

#### Subjective Measures of Physical and Emotional Well-being

The demographic survey was administered via REDCap to understand the participant’s sociodemographic background, physical and mental health history, and current pregnancy-related conditions. Three validated psychological measures that were previously tested with acceptable reliability for studies involving pregnant women were used to assess stress, depression, and anxiety [35-37].

The Perceived Stress Scale (PSS) [38,39] measures the frequency of stress experienced over the past month on a 5-point Likert scale, ranging from 0 (Never) to 4 (Very often). An example of the PSS is “In the last month, how often have you been upset because of something that happened unexpectedly?” The PSS comprised 10 items with scores ranging from a minimum of 0 to a maximum of 40 points.

The Center for Epidemiologic Studies Depression Scale (CES-D) [40] was used to measure depression symptoms on a 4-point Likert scale, ranging from 0 (Rarely or none of the time: less than 1 day) to 3 (Most or all of the time: 5-7 days). An example question of the CES-D is “In the past week, I was bothered by things that usually do not bother me.” The CES-D included 20 items, with scores ranging from 0 to 60 points.

The State Trait Anxiety Inventory (STAI) [41] was adopted to measure the extent of anxiety-related symptoms or emotions on a 4-point Likert scale, ranging from 1 (Not at all) to 4 (Very much). STAI includes 20 items with scores ranging from a minimum of 20 to a maximum of 80 points. An example question of the STAI is “I feel calm at this exact moment.”

A 24-hour recall survey was administered daily in the morning using the THH mobile app to enhance pregnant women’s awareness of personal health. The survey comprised 2 questions: (1) “How would you rate your quality of sleep last night?” with response options 0 (Very poor), 1 (Poor), 2 (Fair), 3 (Good), and 4 (Very good); and (2) How physically active were you in the past 24 hours?” with response options 0 (Not at all), 1 (A little), 2 (Somewhat), 3 (Quite a bit), and 4 (A lot).

The EMA [42] was administrated daily around noontime using the THH mobile app. Its purpose was to enhance the participant’s awareness of her emotional states and to empower her to manage negative emotions by engaging in mindful breathing and physical activity. The EMA included 7 items capturing both positive and negative emotions. Each item score ranged from 0 (Not at all) to 4 (A lot). The questions related to positive emotions assessed the participant's sense of health and safety, with scores ranging from 0 to 8. The questions related to negative emotions assessed the participant's feelings of depression, stress, worry, anger, and loneliness, with scores ranging from 0 to 20.

#### User Experience

User experience was measured using the REDCap closed- and open-ended survey items to assess participant’s experiences with the smart ring, app-based survey, as well as the THH mindful breathing and movement components. An example of a closed-ended survey item was “Practicing THH Mindful Breathing helped me manage stress in my daily life” Response choices were 0 (Completely disagree), 1 (Disagree), 2 (Neither agree nor disagree), 3 (Agree), and 4 (Completely agree). Open-ended questions sought to understand the user’s feedback regarding the THH intervention prototype, for example, “What was your favorite feature? Are there any changes you would suggest for improvement?”

### Analyses

#### Quantitative Analysis

**Figure 2:**
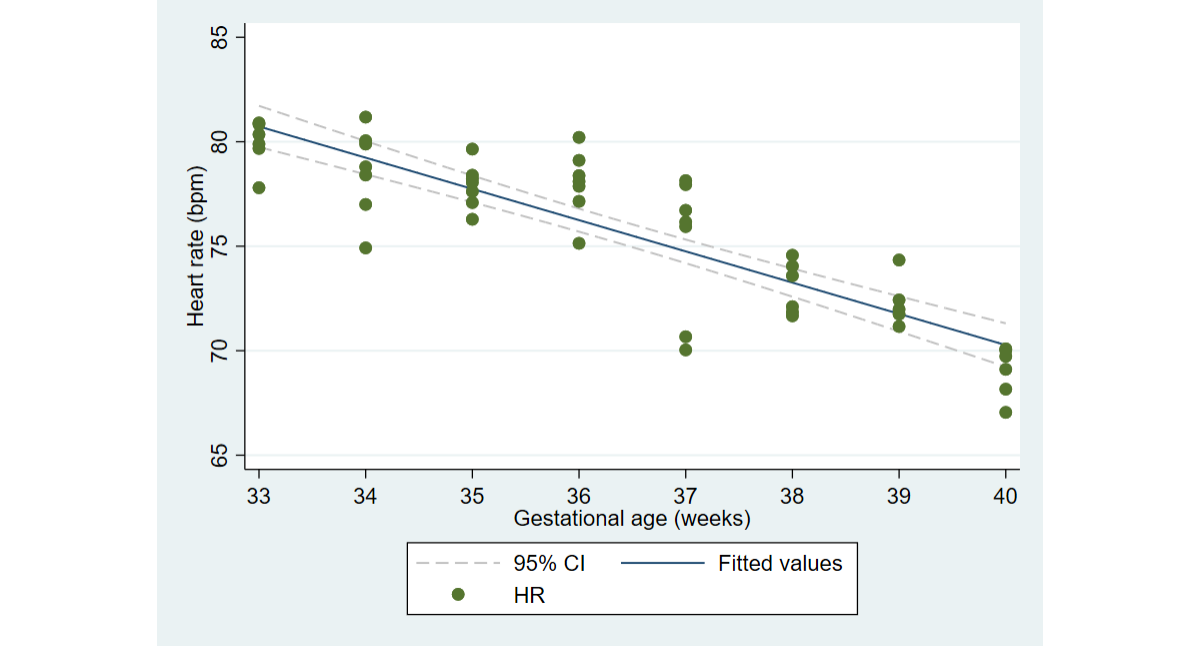
Average resting heart rate. HR: heart rate.

**Figure 3:**
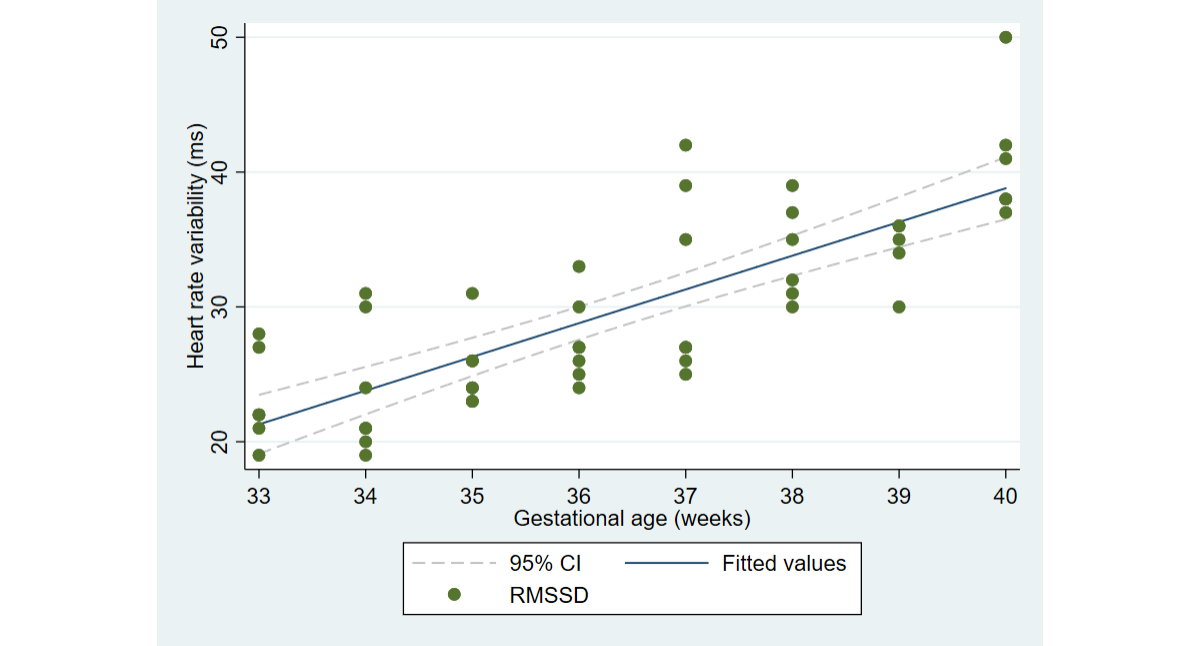
Average resting heart rate variability. RMSSD: root mean square of successive differences.

**Figure 4:**
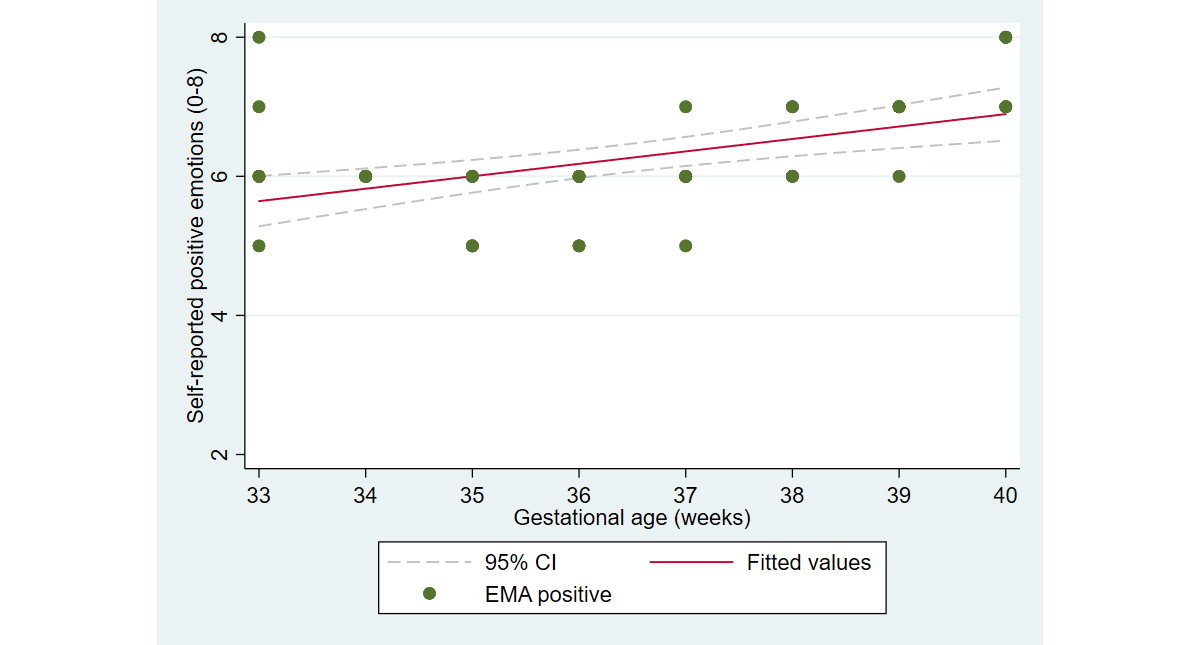
Self-reported positive emotions. EMA: ecological momentary assessment.

**Figure 5:**
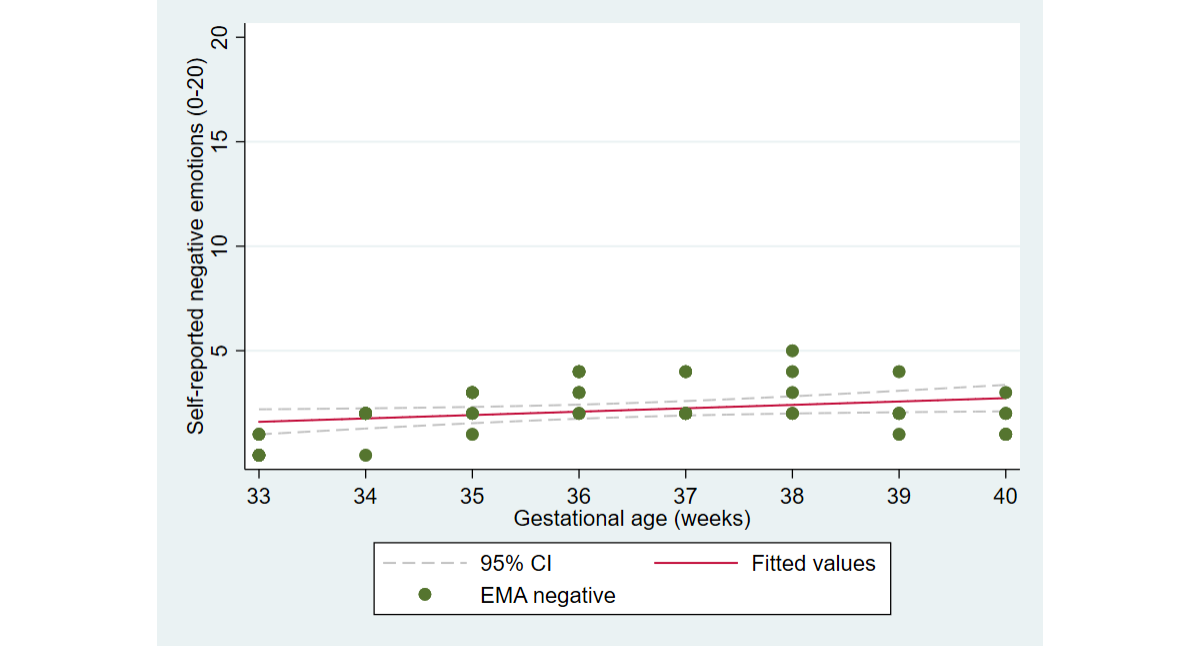
Self-reported negative emotions. EMA: ecological momentary assessment.

**Figure 6:**
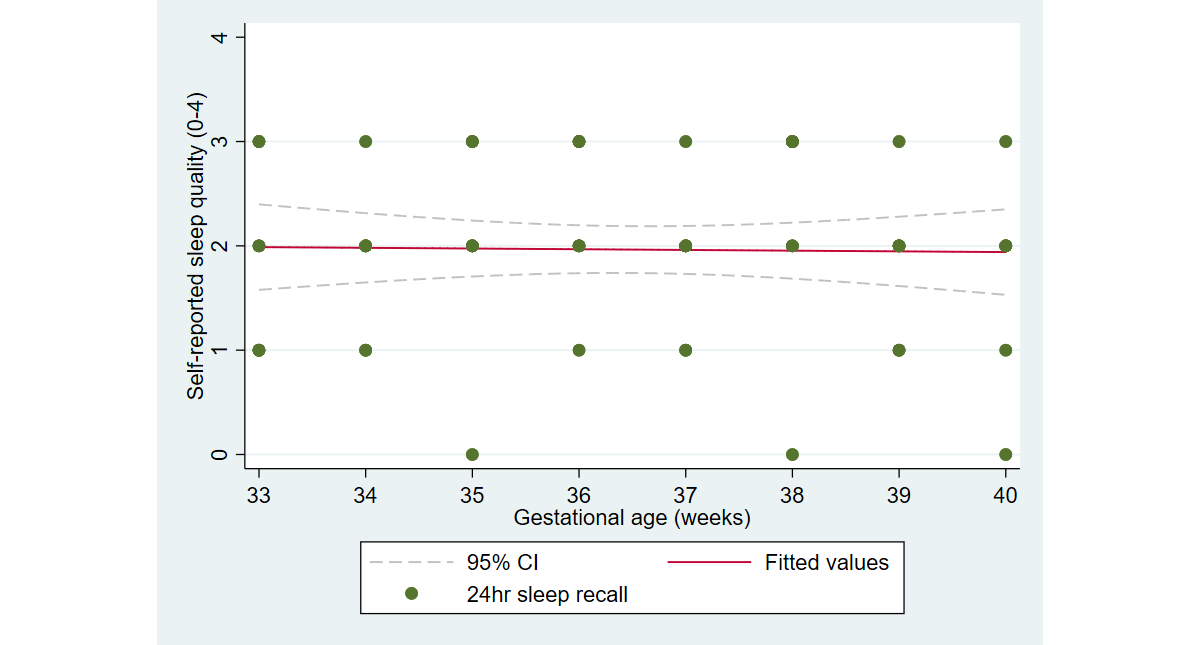
Self-reported quality of sleep.

**Figure 7:**
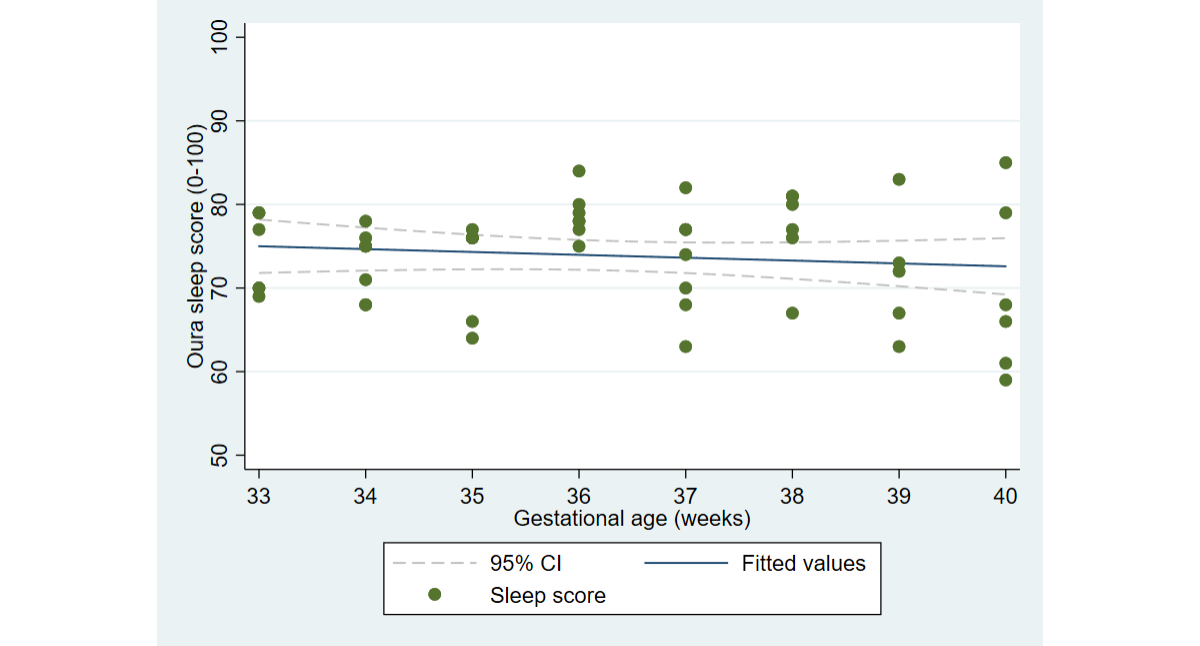
Sleep score as measured by the Oura ring.

**Figure 8:**
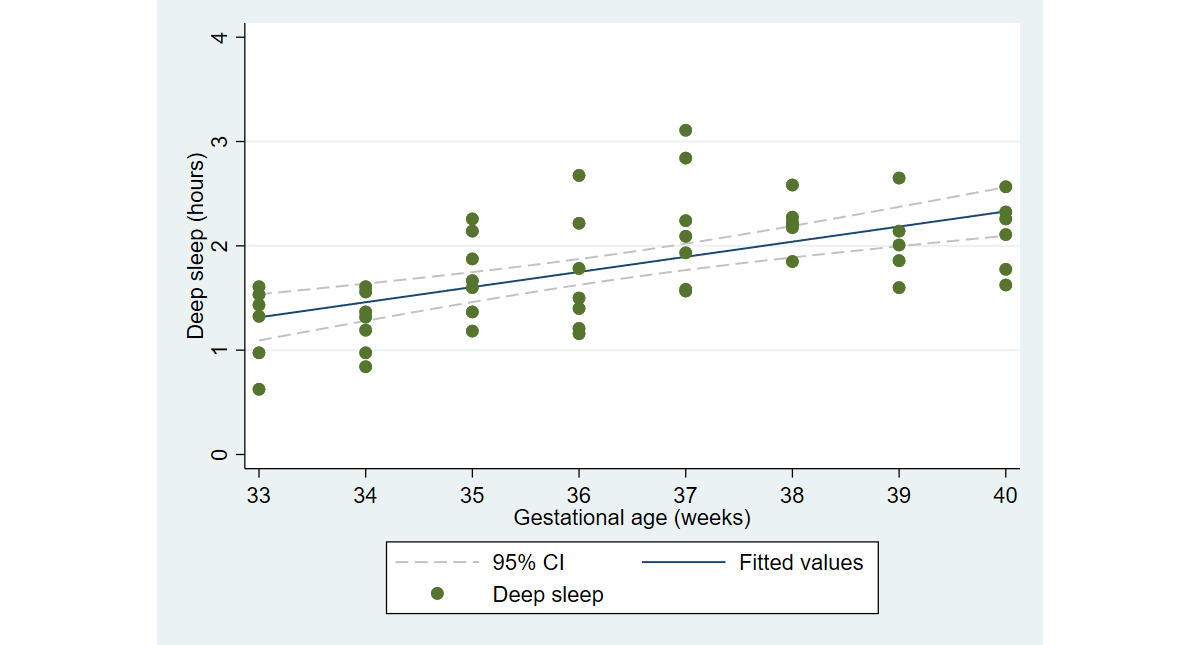
Stage of sleep (deep sleep).

**Figure 9:**
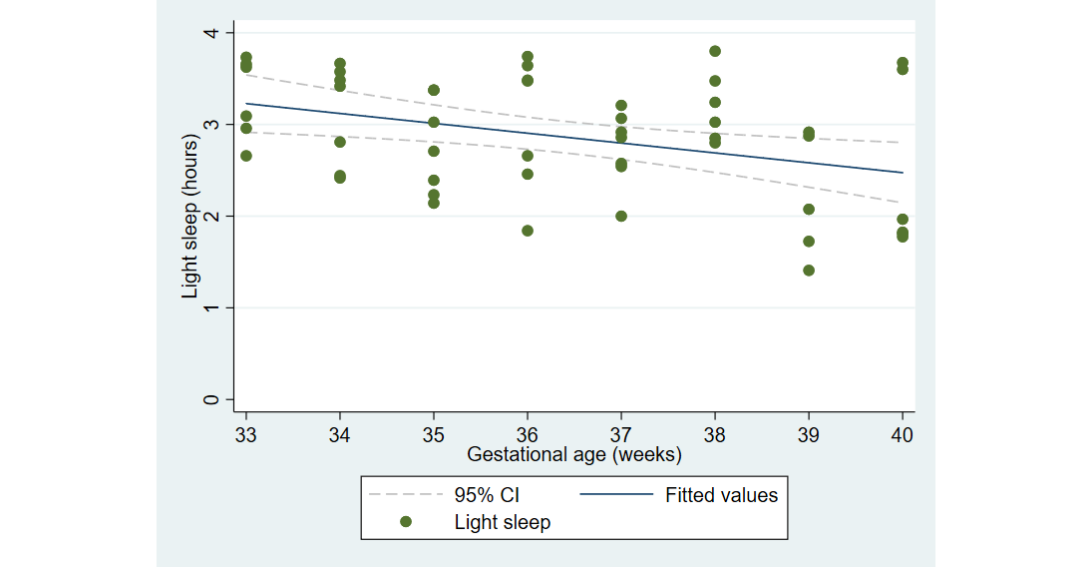
Stage of sleep (light sleep).

**Figure 10:**
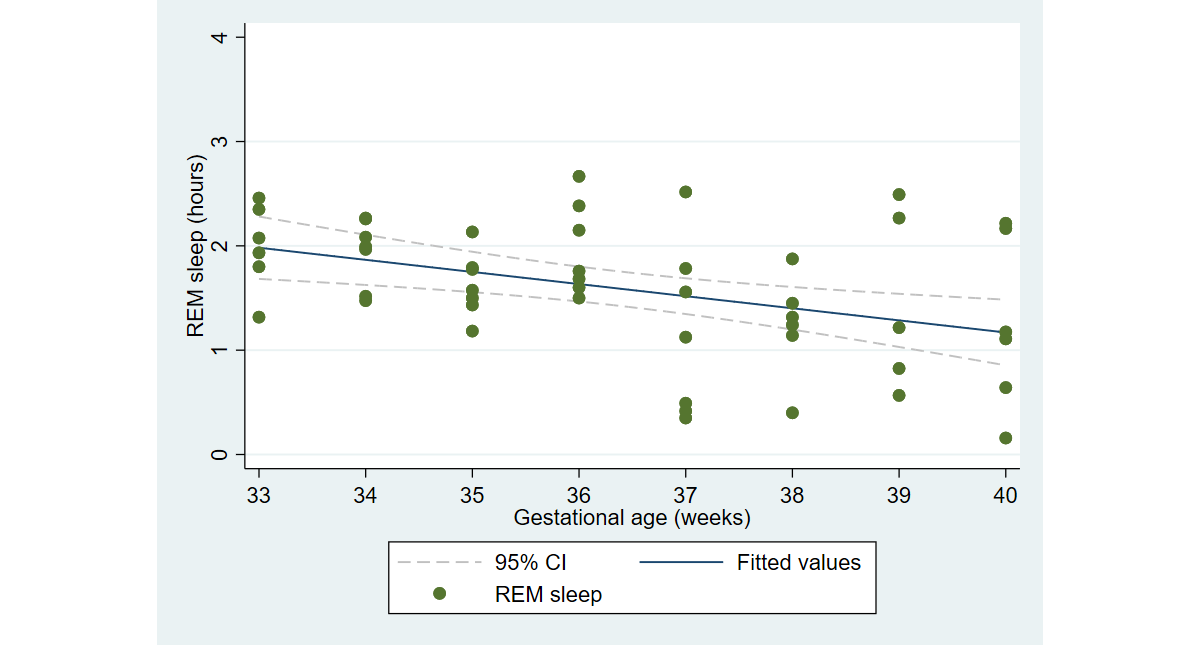
Stage of sleep (rapid eye movement, or REM sleep).

**Figure 11:**
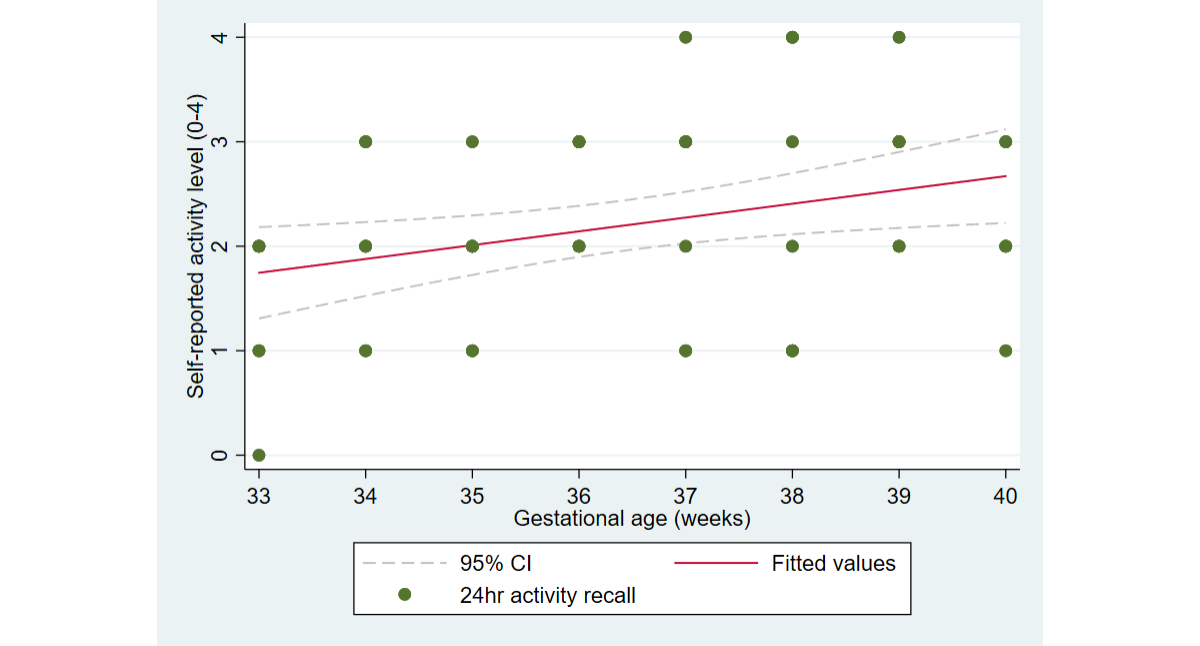
Self-reported level of physical activity.

**Figure 12:**
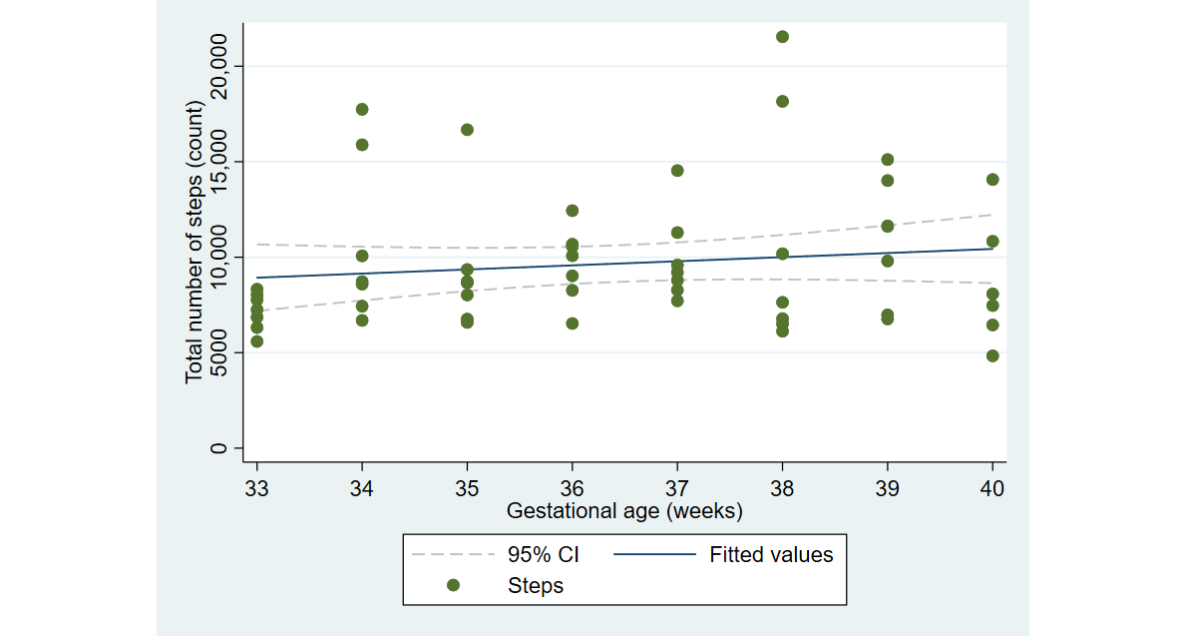
Step recordings as measured by the Oura ring.

Statistical analyses were conducted using Stata software (version 15.1; StataCorp LLC). Normality was determined for the objective and subjective measures using histograms and skewness tests. Regression plots with 95% CIs were used to illustrate objective measures (eg, resting heart rate, resting HRV, sleep score, stages of sleep, and steps) and subjective measures (eg., positive/negative emotions, sleep quality, physical activity level) from 33 to 40 weeks of gestation (Figures 2-12).Given that we sought to understand the correlations between objective and subjective data, subjective data were recoded numerically [43]. Subsequently, Pearson correlation tests were conducted to examine the associations between objective and subjective measures of physical and emotional well-being. The sum scores of the pre-post THH psychological measures were calculated, with reverse coding applied where necessary. A higher PSS, CES-D, and STAI score indicated greater stress, depression, and anxiety level, respectively. The frequency, duration, and completion rate of each THH intervention prototype component were also reported.

#### Qualitative Analysis

For the qualitative analysis, we reviewed the responses to the open-ended questions using content analysis, that is, a research method providing perceived meanings and perspectives [44,45]. Coding and organization of the qualitative data involved 3 steps: (1) review of responses to understand the participant’s perspectives; (2) deriving codes that capture the participant’s experiences; and (3) summarizing codes into themes [44].

### Ethical Considerations

Prior to recruitment and data collection, study approval was obtained from the institutional review board at the University of California, Irvine. The study participant was screened for eligibility and provided with a study information sheet outlining her rights and participation requirements. Data collection began after receiving the participant’s consent.

## Results

### Participant Characteristics

The participant was in her third trimester at the time of enrollment; she was a first-time mother without any medical and pregnancy conditions restricting exercise. She self-identified as Hispanic and Caucasian, preferred to speak English at home, had completed some college education, and worked in an administrative position. At the time of the study, she was enrolled in the Medi-Cal public health insurance plan, was divorced from the father of the baby and living with a male partner. Prior to her pregnancy, she exercised 5 to 6 times a week, but had reduced that to 3 to 4 times during early pregnancy. Factors that motivated her most to exercise included her desire for a healthy baby, healthy pregnancy, and improved mental health. However, she was unable to maintain her regular exercise routine due to the stay-at-home order pertaining to COVID-19 restrictions until she enrolled into the THH intervention. The total duration of her participation in the THH intervention was 8 weeks (ie, 33-40 weeks of gestation). Data were collected from April 18, 2020, to June 12, 2020.

### Quantitative Results

#### Objective and Subjective Measures of Physical and Emotional Well-being

The first aim of the study involved testing the objective and subjective measures of well-being from the third trimester of pregnancy until delivery. Figures 2 and 3 illustrate the cardiovascular response patterns over the course of the final trimester of pregnancy. Decreased resting heart rate was significantly correlated with increased HRV (*r=*–0.92, *P*<.001). Figures 4 and 5 show the positive and negative emotions over time, as reported by the participant. Increased positive emotions were positively correlated with HRV, an objective indicator of emotional states (*r=0*.54, *P*<.001). The correlation between negative emotions and HRV was not significant (*r*=0.18, *P=*.22). Self-reported quality of sleep (Figure 6) was positively correlated with the sleep score generated from the *Oura* ring (*r*=0.52, *P<*.001; Figure 7). In terms of the stages of sleep, deep sleep (Figure 8) appeared to increase, whereas light sleep and REM sleep appeared to decrease (Figures 9 and 10). There was a gradual increase in the level of self-reported physical activity (Figure 11), which was positively correlated with steps (Figure 12) from the smart ring (*r*=0.77, *P*<.001).

#### Experience With the THH Intervention Prototype

The second aim of the study sought to understand the participant’s engagement with the THH intervention prototype and her overall user experience. Table 1 shows the participant’s engagement (ie, frequency and duration) and high completion rate (94.6%-100%) of the THH intervention prototype components, specifically the smart ring, app-based surveys, and mind-body movement..

**Table 1:** Engagement and completion of the Two Happy Hearts (THH) intervention prototype.

THH intervention components and their description			Frequency or duration (% completion rate)
**Smart Ring**			
	Resting heart rate, resting heart rate variability, sleep, step output	Daily for 56 days (100)^a^	
**App-based surveys**			
	24-hour recall of general health	Once a day for 56 days (100)^a^	
	Ecological momentary assessment	Once a day for 53 days (94.6)^a,b^	
**Mind-body movement**			
	Walking (step count)	Average of 9666 steps a day for 55 days (98.2)^a,c^	
	Mindful breathing	Twice a day (3 minutes per practice) for 2 weeks (100)^a^	
	Strength movement	Three times per week for 5 weeks (100)^a^	

^a^Total days in the THH intervention prototype=56 days. Components of the intervention were brought in at different times.

^b^Three days of missing data.

^c^Outlier excluded.

Table 2 shows the observed changes before and after completing the THH intervention prototype. All psychological measures, including PSS, CES-D, and STAI scores, appeared to decrease from baseline to postintervention. The participant rated her satisfaction with the THH intervention an average score of 3.5 (SD 0.9) out of a total of 4 points. She particularly enjoyed using the smart ring, completing the app-based surveys, and mindful strength movement, indicating that the ring was comfortable and easy to use, completing the app-based questions did not interfere with her daily life, and that the strength movement helped her to have a normal delivery.

**Table 2:** Psychological measures before and after the Two Happy Hearts (THH) intervention prototype.

Psychological measures	Baseline score (at 33 weeks of gestation)	Postpartum reduction of score (1 week after delivery)
Perceived Stress Scale (max score = 40)	11	6
Center for Epidemiologic Studies Depression Scale^a^ (max score = 60)	17	7
State Trait Anxiety Inventory^b^ (max score = 80)	24	20

^a^A score of ≥16 indicates depression [46].

^b^A score of ≥45 indicates anxiety [36].

### Qualitative Results

Regarding user experience, the study participant described the intervention as positive and beneficial. Responses to the open-ended surveys were summarized under the 4 main themes described below.

#### Social Support

The participant noted the care and support received from her THH health coach, saying the following:

…feels like talking with a sister or good friend. I knew I was working with someone who genuinely cared, and had my best interests in mind. We worked together for several weeks to better the programming and fine-tune the comfort of the plan I chose.

#### Health Tracking

The THH mobile app was easy to navigate, and the participant enjoyed using the app to practice her exercise routines. The participant also appreciated the ability to self-monitor her sleep and activity level on a daily basis using the smart ring, noting that this function allowed her to effectively make more informed health choices. For example, during one of the sessions with her CHW, she shared that she wanted to be careful “*not birth a ten-pound baby and nine-pound placenta’ and be out of her weight range after delivery*.” Moreover, she valued the helpful information provided by the smart ring, such as suggestions on improving her sleep habits.

#### Well-being and Birth

Our participant stated that she enjoyed practicing the THH strength movement and was confident in her ability to safely engage in the recommended exercise routines. She particularly liked that the mind-body movement helped her to feel more relaxed and sleep better. During labor, she performed 60 squats while having contractions, which she believed helped her to dilate and deliver *quickly.* During the exit interview with her CHW, she stated the following:

I couldn’t have done any of this without the program and probably would have opted for epidural had I not stayed so active...I will continue to implement some of the moves shown to me during my workout...I’ve caught myself taking a moment to breathe and can hear the clip in my head directing me and it has reminded me to pause every once in a while during my crazy busy days.

#### Recommendations

The participant provided some suggestions for improving the study. For example, she noted experiencing some struggle with mindful breathing given that the THH app had only audio cues and no visual cues or animation. She would also have preferred additional calendar notifications for mindful breathing and movement. She suggested that integrating the THH platform with her prenatal health data would enable her to share this information with her health care provider during prenatal visits.

## Discussion

### Principal Results

#### Objective and Subjective Measures of Physical and Emotional Well-being

We were able to use the *Oura* ring to measure objective indicators of physical and emotional well-being from mid–third trimester until delivery. There was a decrease in resting heart rate and an increase in HRV. Deep sleep appeared to increase as light and REM sleep decreased. In addition, we found significant positive associations between self-reported well-being and *Oura* ring measures- higher positive emotions with greater HRV, better sleep quality with a higher sleep score, as well as increased physical activity with a higher step count.

#### Experience With the THH Intervention Prototype

Our participant effectively engaged with the THH intervention as observed by her consistency in wearing the smart ring, completing the app-based surveys, and practicing the mindful breathing and movement routines. Her feedback indicated a positive and rewarding experience with the THH intervention. Specifically, she found it useful to complete the app-based surveys to better understand her physical and emotional well-being, felt confident to practice the strength movement routines safely and attributed her uncomplicated and *quick* labor and delivery to the intervention.

### Comparison With Prior Work

#### Objective and Subjective Measures of Physical and Emotional Well-being

We found that HRV had a significant positive correlation with positive emotions, but not with negative emotions. There are inconsistent findings about these associations in the existing literature. In one study, no significant association was found between fear of childbirth and HRV among pregnant women at 32 to 34 weeks of gestation [47]. However, higher self-perceived stress and lower HRV was reported among pregnant women between 12 and 30 weeks of gestation [48]. The literature also shows mixed results about HRV patterns during pregnancy, with and without intervention [49-51]. It is worth noting that various factors may influence self-reported emotions and HRV, including pregnancy progression, external circumstances such as the COVID-19 pandemic, and different methods of measuring HRV (eg, wearable device in the home setting vs an electrocardiogram in the laboratory), as well as temporal factors taken into account during measurement (eg, continuous vs short-term interval and day vs night) [28,49,50,52,53]. Our findings may have important implications for continuous remote monitoring of objective and subjective emotional health, particularly during heightened levels of stress and anxiety over the course of pregnancy.

With regard to sleep, we observed a decline in rapid eye movement during sleep, as previously reported [5,54]. Deep sleep is an indicator of sleep quality [55]. Interestingly, our study found an increase in deep sleep and a decrease in light sleep. These results are contrary to those of prior observational research involving pregnant women who did not participate in any health intervention during the third trimester [56] and suggest potential benefits of the THH intervention prototype. Additionally, self-reported sleep quality from the app-based 24-hour recall surveys corresponded with the sleep score extracted from the smart ring. Furthermore, self-reported physical activity was consistent with the step count recorded by the ring. Overall, our results suggest that WIoT, such as the *Oura* ring, along with self-reported data from app-based surveys, may provide important objective and subjective measures of physical and emotional well-being during pregnancy.

#### Experience With the THH Intervention Prototype

Recent literature emphasizes the opportunity to leverage technology to promote physical and emotional well-being for pregnant women [57,58]. The high completion rate of the intervention components and positive feedback from our participant shows promise for potential use by underserved pregnant women, many of whom lack access to quality health information, physical activity instruction, stress reduction techniques, and other pregnancy-related resources. Our results align with those of a recent study that found a high adherence rate and acceptance of a mobile health–based physical activity intervention among underserved pregnant women [59]. On the other hand, it is worth noting technology-related challenges that may prevent effective engagement in exercise interventions for pregnant women. For example, time constraints, low technology literacy, and design or complexity of the user interface comprise few factors that have been observed in this study and in previous research [57,60]. Results from our case study are promising, and we anticipate refining the THH intervention to incorporate lessons learned as we support pregnant women to use self-management strategies for improved health and well-being.

### Strengths and Limitations

Our study has several strengths. First is the use of both objective and subjective measures to monitor health and well-being during pregnancy. The *Oura* ring features allowed us to continuously assess important parameters of cardiovascular responses, sleep, and physical activity. In addition, the THH phone app-based surveys captured self-reported health and emotional states daily. Our results suggest the potential of the smart ring and app-based surveys to monitor physical and emotional states. The enthusiasm and commitment demonstrated by the participant’s completion of the THH intervention prototype components was especially valuable given that COVID-19 pandemic regulations made in-person engagement impossible. As seen from her feedback and experience, the intervention provided her with care and support during her pregnancy. Our participant was of low socioeconomic status, and while she was motivated to exercise, THH provided her with the opportunity to do so safely. Therefore, the THH intervention has the potential to reach women in underserved communities, who often face significant barriers to essential health information and feedback about healthy lifestyle choices during pregnancy. It is therefore critical to refine the THH intervention prototype, integrating our participant’s suggestions, as well as test the study’s feasibility in a large sample.

Limitations and possible areas for improvement must be noted. For example, data were available for the third trimester only, which did not allow for comparisons of health measures between trimesters. Recruiting women at earlier stages of pregnancy to examine how physical and emotional health and well-being vary across trimesters is recommended for future research. External factors may have influenced these study results. For instance, the participant was recruited from MOMS OC where she received additional social support during her pregnancy. Also, although the participant desired to have had her final post-intervention interview prior to giving birth, she was unable to complete it until a week following delivery due to time constraints related to birth preparations. Finally, this is a case study; thus, the study results are not generalizable.

### Conclusions

To our knowledge, this study is the first to continuously monitor objective and subjective health and well-being in a pregnant woman from the third trimester until delivery. The consistent patterns between the app-based self-reported data and the *Oura* ring data suggest the potential use of the smart ring to provide valuable health data for pregnant women The enthusiasm and commitment demonstrated by the participant’s completion of the THH intervention prototype was especially promising. It is, therefore, important to refine the intervention and subsequently test its feasibility among underserved pregnant women.

## Abbreviations

ACOG: American College of Obstetricians and Gynecologists
CES-D: Center for Epidemiologic Studies Depression Scale
EMA: ecological momentary assessment
HRV: heart rate variability
PSS: Perceived Stress Scale
REDCap: Research Electronic Data Capture
REM: rapid eye movement
RMSSD: root mean square of successive difference
STAI: State Trait Anxiety Inventory
S&CC: Smart and Connected Communities
THH: Two Happy Hearts
WIoT: wearable internet of things
